# Impact of climate change on the small mammal community of the Yukon boreal forest

**DOI:** 10.1111/1749-4877.12397

**Published:** 2019-10-22

**Authors:** Charles J. KREBS, Rudy BOONSTRA, B. Scott GILBERT, Alice J. KENNEY, Stan BOUTIN

**Affiliations:** ^1^ Department of Zoology University of British Columbia Vancouver British Columbia Canada; ^2^ Department of Biological Sciences University of Toronto Scarborough Toronto Ontario Canada; ^3^ Renewable Resources Management Program Yukon College Whitehorse Yukon Canada; ^4^ Department of Biological Sciences University of Alberta Edmonton Alberta Canada

**Keywords:** community change, long‐term study, population cycles, trophic dynamics, voles

## Abstract

Long‐term monitoring is critical to determine the stability and sustainability of wildlife populations, and if change has occurred, why. We have followed population density changes in the small mammal community in the boreal forest of the southern Yukon for 46 years with density estimates by live trapping on 3–5 unmanipulated grids in spring and autumn. This community consists of 10 species and was responsible for 9% of the energy flow in the herbivore component of this ecosystem from 1986 to 1996, but this increased to 38% from 2003 to 2014. Small mammals, although small in size, are large in the transfer of energy from plants to predators and decomposers. Four species form the bulk of the biomass. There was a shift in the dominant species from the 1970s to the 2000s, with *Myodes rutilus* increasing in relative abundance by 22% and *Peromyscus maniculatus* decreasing by 22%. From 2007 to 2018, *Myodes* comprised 63% of the catch, *Peromyscus* 20%, and *Microtus* species 17%. Possible causes of these changes involve climate change, which is increasing primary production in this boreal forest, and an associated increase in the abundance of 3 rodent predators, marten (*Martes americana*), ermine (*Mustela ermine*) and coyotes (*Canis latrans*). Following and understanding these and potential future changes will require long‐term monitoring studies on a large scale to measure metapopulation dynamics. The small mammal community in northern Canada is being affected by climate change and cannot remain stable. Changes will be critically dependent on food–web interactions that are species‐specific.

### INTRODUCTION

Much of traditional ecological theory is stability-based, and the advent of climate change has forced ecologists to consider the time scale of relative stability in ecosystems. In this paper we report the small mammal community dynamics in a Yukon boreal forest over a time period of 46 years. The boreal forest occupies approximately 57% of the Canadian land surface and is dominated by evergreen coniferous trees, typically white spruce (*Picea glauca*) in the forested valleys of the Kluane Lake area. Common boreal forest trees like black spruce (*Picea mariana*) and lodgepole pine (*Pinus contorta*) are absent in the Kluane Lake area but present in surrounding regions. The ground vegetation in the forested area is comprised of an array of perennial plants of low diversity (Turkington *et al.* 2014). An extensive alpine zone exists above the forested valleys. The herbivorous trophic level in the forested zone is dominated by snowshoe hares (*Lepus americanus* Erxleben, 1777), which fluctuate in a 9–10-year cycle. This cycle affects many, but not all, species in the food web and has been reviewed extensively in Krebs *et al.* (2001, 2018a). We concentrate here on the small mammal community, only one part of the boreal forest fauna described in Krebs *et al.* (2001) and in Boonstra *et al.* (2018).

Beginning with Charles Elton (1942), much research has focused on periodic fluctuations of small mammals in the Northern Hemisphere, which were considered an anomaly in the paradigm of the stability of nature. Much recent work has concentrated on describing the demography of these cycles, identifying populations that do not fluctuate in a regular pattern, and trying to uncover the demographic causes of population changes and the limiting factors behind these changes (Krebs 2013). Of all the possible limiting factors, food shortage, predation, disease and social behavior have been most studied (Boonstra & Krebs 2012; Radchuk *et al.* 2016). Very few small mammal ecologists have concentrated on weather as a direct limiting factor for rodent populations (the exception is Fuller 1969, 1977) on the implied assumption that weather must act through the more immediate factors of food shortage or predation. One consequence of this omission is that if you ask what effect climate change might have on any particular rodent population, there is, at present, little insight. Attention became focused in Europe on the role of climate change when evidence accumulated from long-term studies suggested that populations were showing attenuated cycles, and that possibly small rodent cycles could disappear (Cornulier *et al.* 2013).

Most small mammal studies are short-term, of the order of 3–5 years, yet the importance of long-term studies has been widely recognized (Likens 1989; Hughes *et al.* 2017). In this paper we report on 46 years of population changes in small rodents of the Yukon boreal forest. We use these studies to infer the patterns of change and the potential limiting factors in these populations. Boonstra and Krebs (2006) examined the demography of *Myodes* at Kluane during the Kluane Ecosystem experiments from 1986 to 1996 and suggested 3 hypotheses regarding the causes of population changes in *Myodes*: predation, food and weather. With 20 more years of *Myodes* data we can test these hypotheses.

We wish to answer the following specific questions for small rodents in the Yukon boreal forest:

1. Do populations of the common species fluctuate periodically in 3–4-year cycles?
2. Do populations of the different species fluctuate in phase?
3. Are these population fluctuations disappearing as a result of climate change?
4. What ecological factors involving predation, food supply and/or weather drive these demographic changes?
5. Does the dominant 9–10-year cycle of snowshoe hares affect small rodent population dynamics?

### MATERIALS AND METHODS

Ten small rodent species occur in the Kluane region. Red-backed voles [*Myodes rutilus* (Pallas, 1779)] are the most common, comprising approximately 70% of the biomass of this group. Deer mice [*Peromyscus maniculatus* (Wagner, 1845)] and 4 species of voles (*Microtus* spp.) are less abundant. In addition, there are 4 rare species present but rarely caught (Krebs & Wingate 1976), as well as chipmunks (*Tamias minimus* Bachman, 1839), and shrews on which we have inadequate data. Monitoring for changes in the abundance of small mammals in the Kluane region has been carried on annually since 1973. Many of the details of these methods are described in Boonstra *et al.* (2001). From 1973 to 1975 we sampled widely throughout the Kluane valley with Museum Special snap traps and on 4 live-trapping grids with Longworth live traps. Our early studies with snap trapping demonstrated that voles and mice in the Kluane area showed spatial synchrony (Krebs & Wingate 1976, 1985). After 1975, virtually all our data came from live trapping standard grids (10 by 10 with 15-m spacing [2.3 ha] with either 50 or 100 Longworth live traps). We used a variety of sites in the area just south of Kluane Lake for live trapping in the first 10 years of our studies, but by 1979 we had begun trapping Grid J, one of our standard control grids for small rodents, and it has been trapped continuously ever since. We set up live trapping grids in 1973 at Mile 1050 of the Alaska Highway, and this grid was shifted slightly to become a standard control grid (Silver) in 1987. We have put all the available data together here from all our live trapping to obtain a comprehensive view of small mammal population changes, fully realizing that the data are incomplete for several small mammal species that are present but rare. There are 3 habitats that consist of over 90% of the landscape of the Kluane region: closed spruce forest, open spruce forest, and willow shrub. These are intermingled at a fine scale and we found in our early work from 1973–1975 that all 3 habitats contained a similar small mammal fauna. We set up sampling grids at convenient locations in open and closed spruce forest, which are the 2 most common habitats in the valley. All the 3 major habitats are a product of fires 150 to 300 years ago. We were not able to sample any of the rare habitats in the Kluane region, such as damp habitats along water courses and ponds, and we have not included samples taken in the alpine above treeline.

Live-trapped rodents were typically sampled in 2-day sessions with 3 trap checks and whole oats & apple as bait in Longworth live traps. We trapped after snow melt in spring at the start of the breeding season (early May) and in early autumn (September) typically at the end of breeding. We used Efford's spatially-explicit capture–recapture program (SECR, Efford & Fewster 2013) to estimate density except when the number of animals captured was 4 or fewer when we used the minimum number known alive and the effective trapping area of our grids (2.8 ha). Trappability was very high for all the major rodent species. We calculated 2 indices of population change (summer growth and overwinter decline) using live-trap data. Summer population growth was the Fall estimate ($t$)/Spring estimate ($t$) and overwinter population decline was the Spring estimate ($t + 1$)/Fall estimate ($t$).

To estimate the potential food supply for small rodents we counted ground berries in the boreal forest at 10 locations every summer from 1995 to 2018. Five major ground shrubs produce ground berries in the Kluane forest: bearberry (*Arctostaphylos uva-ursi*), red bearberry (*A. rubra*), cranberry (*Vaccinium vitis-idea*), crowberry (*Empetrum nigrum*) and soapberry (*Shepherdia canadensis*). Toadflax (*Geocaulon lividum*) was also present in many locations. We monitored the production of ground berries annually on an average of 707 40 × 40-cm fixed quadrats (Krebs *et al.* 2009).

We do not have data on the abundance of bird predators for our sites for most of our monitoring years, and this is a large gap in our attempts to determine the impact of bird predation on small rodents in this ecosystem. Our studies on raptor predation extended only from 1987 to 1996 and are reported in Doyle and Smith (2001). The most extensive data available from these studies was on the great-horned owl (*Bubo virginianus* Gmelin, 1788).

We have constructed energy flow estimates for the most common mammals and birds of the Kluane area from equations for energy use given in Nagy *et al.* (1999) and estimates of average density over 10 years from 2 periods, 1986–1996 and 2003–2014. We appreciate that these energy pyramids are only approximate and represent a 10-year average, but they illustrate in an important way how the terrestrial vertebrate community at Kluane has changed over time.

Two small mammalian predators – marten [*Martes americana* (Turton, 1806)] and ermine (*Mustela erminea* Linnaeus, 1758) – are established in the Kluane region but vary greatly in their abundance. Both species are major predators of small rodents. Ermine were caught in our live traps, but not frequently. A small number of marten ($n$ = 30) were transplanted to the Kluane area in 1984–1987 to augment the resident, low-density population (Slough 1989). While initial survival of transplanted marten was high, the success of this small-scale effort in effectively augmenting the resident population is unknown. The least weasel (*Mustela nivalis* Linnaeus, 1766) has been caught in the Kluane area but is rarely present. One was captured by hand over the period 1986–1996, and we did not catch any in annual live trapping from 1986 to 2018.

Estimates of terrestrial predator abundance in the Kluane area have been indexed by snow tracking (O'Donoghue *et al.* 2001). These snow track transects have been carried out every year since 1986 along the same 25-km transect. Snow tracking data estimate activity that we assume to be related in some approximate way to population abundance. It is possible that ermine abundance is underestimated if they do not enter Longworth traps often. Snow tracking in the autumn when minimal snow is present should give us a reliable index of abundance, but we cannot yet translate that to absolute abundance.

Weather changes in the Kluane area have been recorded by the Haines Junction Meteorological Station of Environment Canada since 1945 and provide a general background for the observed biological changes. In addition to the standard measurements of temperature, rainfall and snow depth, since 2004 we have measured temperature profiles in winter using I-buttons

(Maxim Thermochron iButtons DS 1921G-F5) mounted on 2 replicate wood stakes with 9 buttons ranging from ground level to 80-cm high. I-buttons were set to record temperature at 4-hour intervals year-round.

Statistical analyses were carried out in NCSS 10 (Number Crunching Statistical System, https://www.ncss.com). Multiple regression models to investigate hypotheses about the relative importance of weather, predation and food factors were first investigated with All Possible Regressions in NCSS 10 and then tested with Robust Regressions, following the general recommendations of Mac Nally (2000). When several models seemed of equal value, we used $AIC_c$ to determine the best model following the methods of Anderson (2008). For all food-related statistical models we tested current annual production as well as that of the previous summer for possible predictive value because some berries carry over from one year to the next.

### RESULTS

#### Climate change in the Kluane Region

Winter temperatures (November to April) have been increasing on average since 1970 by 0.47 °C per decade (see Fig. 2 in Boonstra *et al.* 2018). Summer temperatures during the growing season are increasing more slowly, at 0.26 ºC per decade (Fig. 1). Summer rainfall (June to August) is highly variable and over the 1970 to 2018 period shows no significant change with a high coefficient of variation (CV = 40%). The net result is a slowly changing climate with temperatures increasing in both winter and summer and unpredictable fluctuations in summer rainfall. The rate of temperature change at Kluane (2 °C per 50 years on average) is very high by global standards of changing temperatures.

#### Mammal community changes

The overall change in the composition of the herbivore community at Kluane over the past 30 years is illustrated in Fig. 2. We averaged the best estimates of the population size of the 7 most common forest herbivore species, ignoring the species with low abundance in this part of the boreal zone. We converted these average population estimates to energy use with the equations provided in Nagy *et al.* (1999). If we use estimates of standing biomass, we obtain a similar picture (Boonstra *et al.* 2016), but we consider that energy use is a better estimate of ecosystem composition. There are 3 major differences in the herbivore community over this 30-year period. First, the contribution of small rodents to energy flow in the herbivore community increased from 9% in 1986–1996 to 38% in 2003–2014. This change is the major topic of this paper. Second, arctic ground squirrels [*Urocitellus parryii* (Richardson, 1825)] declined 95%, changing status from very common to rare over this period (Werner *et al.* 2015). Third, snowshoe hares decreased 55% in energy flow in the decade from 1986–1996 to 2003–2014, associated with lower average densities after 2000 (Krebs *et al.* 2018a). Thus, the overall energy use of mice and voles has increased 73% in the period from 1986–1996 to 2003–2014. We address the possible reasons for this change in mouse and vole populations in this paper.

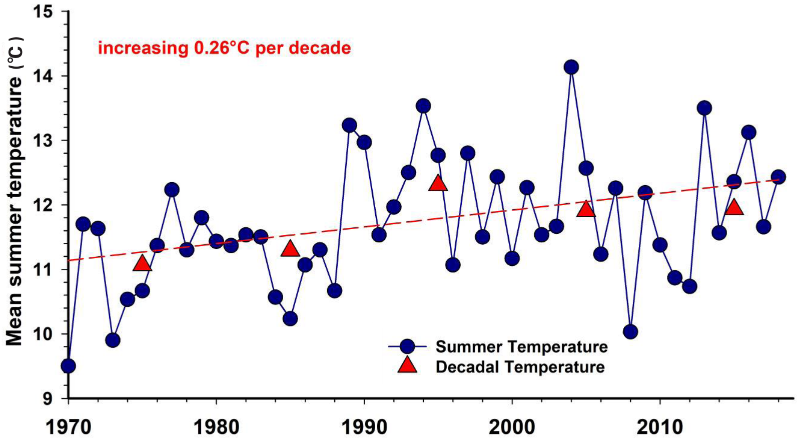

**Figure 1** Summer growing season average temperatures, 1970–2018, from Haines Junction Weather Station. The overall regression is: Summer temperature = −40.28 + (0.0261 × year), $n$ = 49, $R^2$ = 0.14, CV = 7.9%. Summer is June 1 to August 31.

### Changes in mice and vole population abundance

Four rodent species in this boreal forest are rarely seen or caught: *Phenacomys intermedius* Merriam, 1889, *Zapus hudsonius* (Zimmermann, 1780), *Lemmus sibiricus* (Kerr, 1792) and *Synaptomys borealis* (Richardson, 1828). These species are nearly impossible to study because they are so rare, and we have little information on their ecology in this ecosystem. Their trappability may be extremely low relative to the main species but we know they are rare from both live-trapping and snap-trapping studies (Krebs & Wingate 1976). We can only say that in terms of energy flow they are negligible.

The relative abundances of the most common species of small mammals has changed dramatically during the last 45 years (Fig. 3). In the 1970s, the deer mouse *Peromyscus* and the red-back vole *Myodes* were the 2 dominant species, each comprising approximately 40% of the rodent numbers. By the 2000s dominance had switched so that *Myodes* comprised approximately two-thirds of the rodent numbers and *Peromyscus* only 12%. The 4 *Microtus* species, by contrast, have not changed very much, being consistently as a group comprising approximately 20% of the numbers of small mammals.

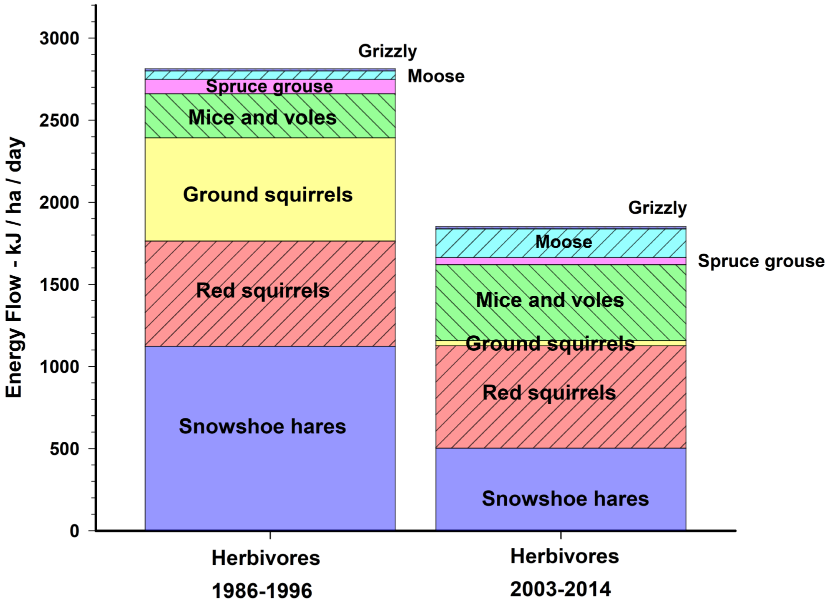

**Figure 2** Energy flow averages for the mammalian herbivores in the Kluane boreal forest ecosystem for the periods 1986–1996 and 2003–2014. Both these periods equally span a full 10-year snowshoe hare cycle from the beginning of 1 cycle to the beginning of the next. Energy flow estimated from average density of each species and the equations in Nagy *et al.* (1999).

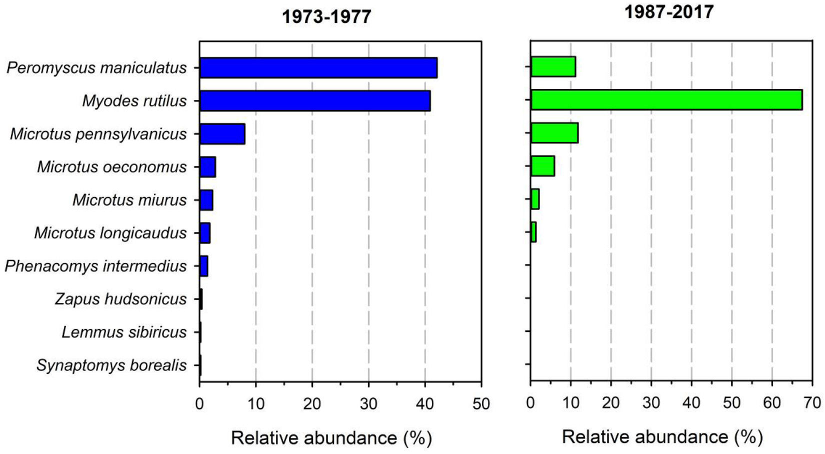

**Figure 3** Relative abundance of 10 species in the small mammal community at Kluane from the start of our studies (1973–1977) to the more recent studies. The period 1978 to 1986 is not included because we did not have adequate comparable data on the less common species of small mammals for that time period.

*Red-back voles*

Figure 4 shows the changes in the abundance of the red-back vole *Myodes* from 1973 to 2018. There is a clear 3–4-year cycle in this species in the Kluane area (spectral density peaks at 3.67 years). There is a suggestion of an increase in amplitude of peak densities over time. A linear regression with peak year as the independent variable and peak density as the dependent variable is suggestive but not statistically significant. A clearer 2 sample *t*-test with a division point at the year 2000 is highly significant. From 1973 to 1999, 7 peaks averaged 9.7 *Myodes* per ha, while from 2000 to 2018, 5 peaks averaged 19.5 *Myodes* per ha. Significance values are meaningless for these comparisons since they were selected by inspection of Fig. 4, but the effect size is large, a doubling of peak *Myodes* density after 2000.

Marten were nearly absent at Kluane before the year 2000 (9). Their arrival coincided with the break point in peak abundance of *Myodes*, but the surprise was that increased predator abundance coincided with an *increase* rather than a decrease in *Myodes* abundance. Ermine abundance as indexed by snow tracking also increased after 2000 (Fig. 5). There was no association between ermine abundance and marten abundance from 1986 to 2000 (because marten were virtually absent) but a high correlation ($r = 0.74$) from 2000 to 2018. If predation is a significant variable affecting rodent numbers, *Myodes* density should have declined after 2000 but, instead, it increased. This suggests that rodent numbers drive predator numbers but that the reverse effect is not as strong.

Winter snow conditions could also be related to *Myodes* demography. However, neither maximum snow depth nor average levels of winter snow were closely related to overwinter decline of *Myodes* ($r_s = -0.10$ and 0.09, $n = 45$ years). More detailed local snow data from 2000 to 2018 shows the same result (Fig. S1), at best a weak positive relationship between maximum snow depth in winter and winter population changes in *Myo-*

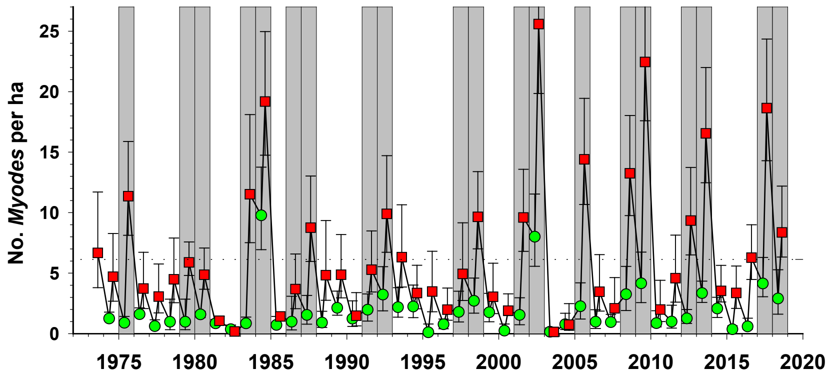

**Figure 4** Spring and fall density estimates for the red back vole *Myodes rutilus* at Kluane, 1973 to 2018. Spring = green circles; fall = red squares. Peak years are shaded in grey. Year tics are 1 January of each year. The horizontal dashed line is the average density of *Myodes* over the entire time period (6 per ha). Confidence intervals are 95%.

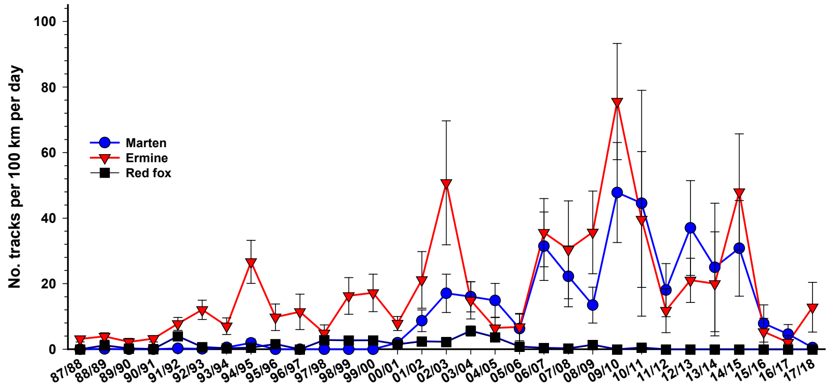

**Figure 5** Snow track indices of small mammal predators at Kluane, 1987 to 2018. Marten became abundant only after 2000, and at the same time ermine increased greatly in abundance. Red foxes have never been common in the Kluane boreal forest. Error bars are 95% confidence limits.

*des*. We have no evidence that snow depth in winter plays a strong role in determining the amount of population change of *Myodes* over the winter non-breeding period.

By measuring winter temperature at ground level in winter, we could ask if winter severity measured by daily temperatures below −10ºC independent of snow depth could be related to overwinter disappearance of *Myodes.* We began these measurements only in 2004 and with 14 years of data we found no relationship between winter temperature severity at ground level and *Myodes* overwinter disappearance ($r = -0.09$). Whatever the cause of the variation in *Myodes* overwinter declines, it does not appear to directly simply relate to the winter temperature under the snow at ground level. There was no sign of ground level icing events in our area, although some mid-to-late winter rain-on-snow events did occur.

We combined all the measured variables into 1 multiple regression analysis to measure the relative strength of food and predator numbers on both summer growth and winter declines of *Myodes.* For summer growth, the major predictors were the berry crops of *Empetrum nigrum*, *Vaccinium vitis-idaea*, *Geocaulon lividum* and *A. uva-ursi* in the current year (Table 1). For winter declines, the major predictors were the *E. nigrum* and *A. rubra* crops of the previous summer and the abundance of ermine and marten in the current winter (Fig. 6, Table 1). Thus, both food supplies and predator numbers, but not snow conditions or winter temperature, affect population changes in *Myodes.*

*Deer mice*

Deer mice (*Peromyscus*) now make up a small part of the biomass of the small rodents at Kluane, approximately 10%. Their population changes have been inexplicable for us (details in Krebs *et al.* 2018b; see Fig. S2 in Supplementary Materials). We have carried out a detailed statistical analysis of our deer mouse data. Sum-

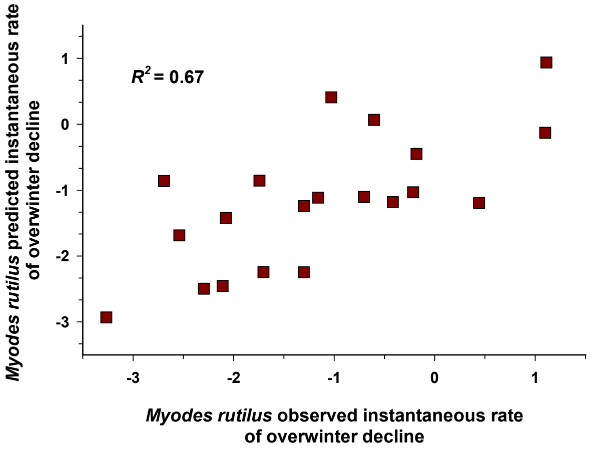

**Figure 6** Prediction of red-back vole *Myodes* overwinter decline in relation to observed winter decline. Prediction was achieved by the equation given in Table 1 using crops of berries the previous summer, 1997 to 2018, ermine and marten abundance. Berries included *Arctostaphylos rubra* and *Empetrum nigrum*. Overwinter decline was calculated as the instantaneous rate of spring density in year $t$ + 1/fall density in year $t$.

**Table 1** Stepwise predictions of *Myodes rutilus* summer population growth and winter population declines

| Predictive variable | Multiple regression | Sample size (years) | $R^2$ | Variables considered but omitted |
|---|---|---|---|---|
| Summer population increase | Summer increase† = 9.63 + 0.1074 *Vaccinium* − 0.1078 *Empetrum* – 0.2405 *Geocaulon* | 22 | 0.58 | May, June, July and August temperature, *Arctostaphylos uva-ursi*, *Arctostaphylos rubra*, *Shepherdia canadensis*, Mushroom crop, ermine and marten abundance in previous winter |
| Winter population decline | Overwinter_change† = −0.670 + 0.0537 × *Empetrum*‡ − 0.1757 × *A. rubra*‡ − 0.0888 × Ermine + 0.0685 × Marten | 21 | 0.67 | Winter temperature, winter average snow depth, winter maximum snow depth, previous summer (*Arctostaphylos uva-ursi*, *Shepherdia canadensis*, mushroom crops) |

All possible regressions were computed in NCSS 10 to determine the optimal number of variables for prediction using Mallow's $C_p$ followed by stepwise regression to select the relevant variables, followed by robust multiple regression in NCSS 10. †Summer increase = fall density/spring density. Overwinter change = $\log_e$(spring density of $t$+1/fall density of year $t$). ‡Berry production of previous summer.

mer data on the berry crops of *A. uva-ursi* and *Geocaulon lividus* along with snow tracks of ermine in the previous winter could predict summer increases in deer mice. However, we could find no correlation with temperature, snow levels, or the abundance of ermine or marten that could predict the size of the winter decreases over the 19 years for which we have detailed data (Table 2). Since 1995, deer mice have never exceeded 4 animals per ha, a density so low that predators might not make a living hunting them and diseases might not easily transmit among them (see Luis *et al.* 2012). We have found no evidence of hantaviruses in Kluane *Peromyscus* and it is unlikely that these virus diseases could transmit in our populations with such a low density.

#### *Microtus voles*

The 4 *Microtus* species that are found at Kluane present yet another enigma to our understanding. Figure 7 shows population trends in this species group over the past 33 years, and Table 3 gives the predictive regressions for this species group. The clear 3–4-year population cycles are expected from these meadow voles, but the unusual pattern shown is that the dominant species at the peak changes from cycle to cycle, even though we are live trapping on exactly the same areas over this time period. The minor *Microtus* species do not disappear but in the peaks 95–100% of the captures are the dominant species (Fig. 7). *Microtus oeconomus* (Pallas, 1776) and *M. pennsylvanicus* (Ord, 1815) can be

**Table 2** Stepwise predictions of *Peromyscus maniculatus* summer population growth and winter population declines

| Predictive variable | Multiple regression | Sample size (years) | $R^2$ | Variables considered but omitted |
|---|---|---|---|---|
| Summer population increase | Summer_increase† = 3.686 + 0.0706 × *Arctostaphylos uva-ursi* − 0.0982 × *Geocaulon* − 0.0348 × Ermine | 19 | 0.74 | May, June, July August temperature, *Empetrum nigrum*, *Arctostaphylos rubra*, *Shepherdia canadensis*, *Vaccinium vitis-idaea*, mushroom crop, marten abundance in previous winter |
| Winter population decline | No predator or food supply or temperature variables could predict overwinter decline for *Peromyscus* | 19 | 0.19 | Ermine and marten abundance, winter temperature, winter average snow depth, winter maximum snow depth, previous summer (*Empetrum nigrum*, *Arctostaphylos rubra*, *Arctostaphylos uva-ursi*, *Shepherdia canadensis*, mushroom crops) |

All possible regressions were computed in NCSS 10 to determine the optimal number of variables for prediction using Mallow's $C_p$ followed by stepwise regression to select the relevant variables, followed by robust multiple regression in NCSS 10. †Summer increase = fall density/spring density. Overwinter change = $\log_e$(spring density of $t$ + 1/fall density of year $t$).

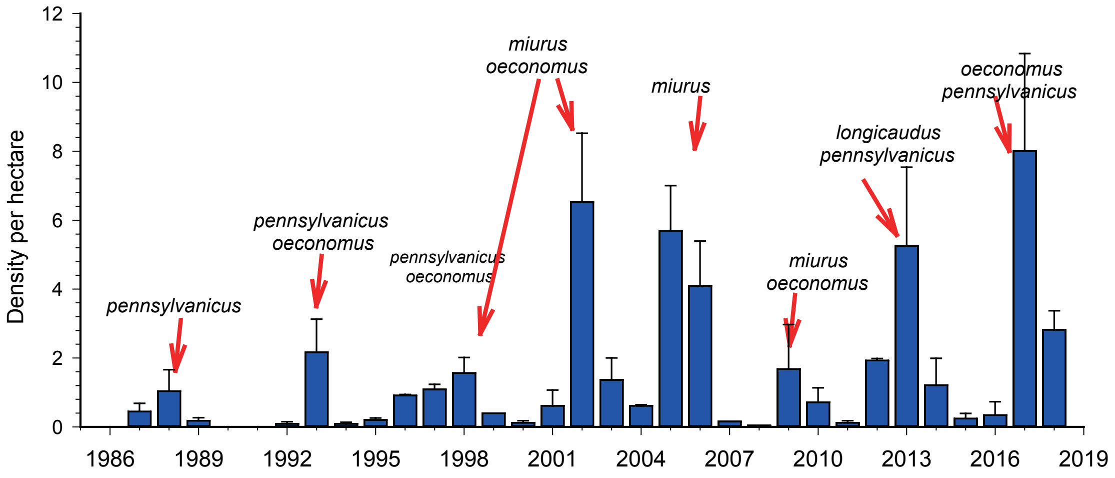

**Figure 7** Autumn population density of *Microtus* spp. in the boreal forest at Kluane, 1986 to 2018. A 3–4-year cycle is clear, but the species present at the peak changes over time. Error bars are 95% confidence limits. (Updated from Fig. 6 in Krebs *et al.* 2018b with permission.)

difficult to tell apart in the field (Boonstra *et al.* 2001) and the peak densities of 1993, 1998 and 2017 involved a mixture of these 2 *Microtus* species. The peak abundance of *Microtus* has also increased since 2000, like the pattern found in *Myodes* (Fig. 4).

Why should this species replacement pattern occur? We can find no one who has studied voles who has described such a pattern of cyclic species replacement. There are 2 possible models of how this pattern could arise. First, there may be a competitive dominance hierarchy among these species. We do not know if there is direct interference competition between any of these species, except for our studies on *M. miurus* (Osgood, 1901) and *M. oeconomus*, in which we could demonstrate no competitive release when we removed one of them and observed the response of the other (Galindo & Krebs 1985a,b). We could see no clear patterns of species interference in this small rodent community. If there was a size dominance in the *Microtus* guild, *M. longicaudus* (Merriam, 1888) (average adult weight 50 g) should win. The average weight of the other *Microtus* species ranges from 25 to 32 g. A second model to explain Fig. 7 could be random species replacement in a patchy network. All these *Microtus* prefer grassland, which occurs in small patches throughout the Kluane boreal forest. If populations go locally extinct in the low phase of these 3–4-year cycles, the next colonizer could become the winner for the next peak. Such metacommunity dynamics (Holyoak *et al.* 2005) implicates an array of possible mechanisms from dispersal dynamics to random patch extinction among interacting species within a community. Much more experimental work and landscape analyses will be needed to determine if this explanation fits this *Microtus* community. The challenge this suggestion faces is that of doing experiments with the very low densities of these *Microtus* species (except for the peak years).

We come away not understanding the processes driving these changes: in the end we have a group of *Microtus* species who appear to have little direct interactions through interference competition yet persist with different habitat requirements and possible food differences.

### Changes in predator populations

The major predators of all these small rodents are ermine and marten, as well as the great horned owls [*Bubo virginianus* (Gmelin, 1788)], the boreal owl [*Aegolius funereus* (Linnaeus, 1758)], the northern hawk owl [*Surnia ulula* (Linnaeus, 1758)], and a variety of other raptors (Doyle & Smith 2001). For the 1986–1996 period, we have very good quantitative data on bird predators to correlate with the changes in vole and mice numbers (Doyle & Smith 2001; Rohner *et al.* 2001), and we were able to continue these observations only for great-horned owls until 2010. The overall mammalian predator energy flow period has changed dramatically during the past 40 years. Marten accounted for 0.1% of the energy flow in the 1986–1996 period and then increased to account for approximately 7% of the energy flow from 2003 to 2014. Over this same time period, the contribution of ermine to overall predator energy flow tripled. Figure 8 shows the overall average contribution to energy flow in the predator populations for which we have good data. There is a suggestion that over this 30-year time period predation rates might have increased on small rodent populations.

**Table 3** Stepwise predictions of *Microtus spp.* summer population growth and winter population declines. Berry production of the previous summer was used for each berry species as a possible predictive variable for winter population changes

| Predictive variable | Multiple regression | Sample size (years) | $R^2$ | Variables considered but omitted |
|---|---|---|---|---|
| Summer population increase | Summer_increase[†] = 7.593 + 1.5532 × Previous spring density − 0.3148 × *Geocaulon* − 0.1604 × Ermine + 0.2781 × Marten | 21 | 0.59 | May, June, July, August temperature, *Empetrum nigrum*, *Arctostaphylos uva ursi*, *Arctostaphylos rubra*, *Shepherdia canadensis*, *Vaccinium vitis-idaea*, mushroom crop |
| Winter population decline | Winter_decline = −0.5562 + 0.02743 × *A. uva-ursi* + 0.02169 × *Shepherdia* − 0.1053 × *Geocaulon* − 0.02820 × Ermine | 19 | 0.19 | Marten abundance, winter temperature, winter average snow depth, winter maximum snow depth, previous summer (*Empetrum nigrum*, *Arctostaphylos rubra*, *Vaccinium vitis-idaea*, mushroom crops) |

All possible regressions were computed in NCSS 10 to determine the optimal number of variables for prediction using Mallow's $C_p$ followed by stepwise regression to select the relevant variables, followed by robust multiple regression in NCSS 10. [†]Summer increase = fall density/spring density. Overwinter change = $\log_e$(spring density of $t + 1$/fall density of year $t$).

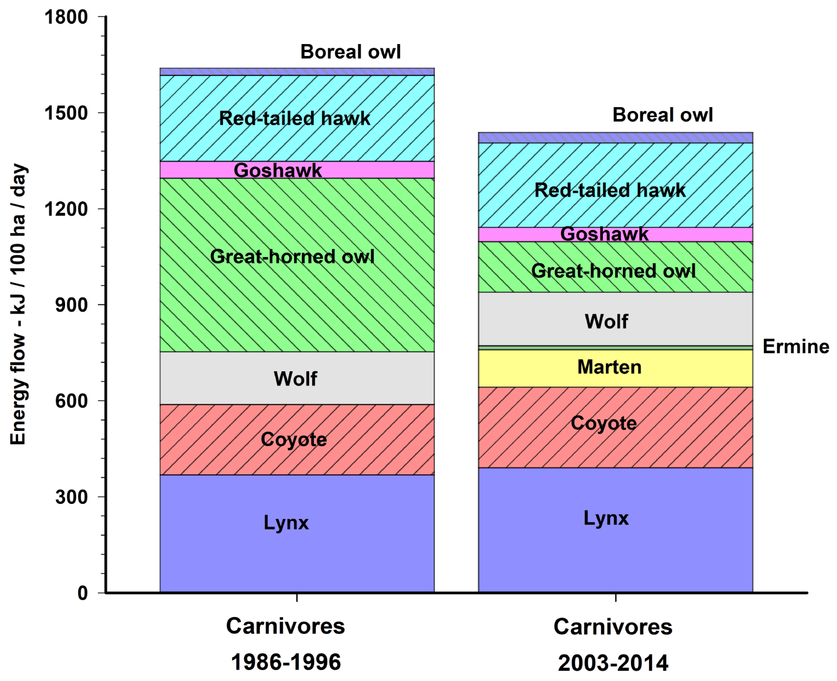

**Figure 8** Energy flow averages for the mammalian carnivores in the Kluane boreal forest ecosystem for the periods 1986–1996 and 2003–2014. Both these periods contain a full 10-year snowshoe hare cycle. Energy flow estimated from the estimated average density of each species and the equations in Nagy *et al.* (1999). The small band between marten and wolf is the energy flow to ermine.

## DISCUSSION

Given these data, we can answer the 5 questions posed in the Introduction.

1. Populations of *M. rutilus* and *Microtus* spp. fluctuate periodically in 3–4-year cycles, but *P. maniculatus* does not. *M. rutilus* has been challenged in some short-term studies as a non-cyclic vole in the boreal zone of North America (e.g. Boonstra & Krebs 2006, 2012), but this conclusion does not fit our long-term Yukon data, which is clearly cyclic. The closely-related *Myodes glareolus* in Fennoscandia is clearly cyclic in that region, and we would predict that *M. rutilus* is cyclic across the boreal forest zone of North America. *P. maniculatus* has never been observed to be cyclic so this conclusion is not new.

2. Populations of the 3 major species at Kluane are only weakly correlated. Autumn population densities of *Myodes* and *Microtus* are significantly correlated ($r$ = 0.77) but neither of these is correlated with *Peromyscus* fall numbers ($r$ = 0.26 to 0.35). We suspect this correlation of *Myodes* and *Microtus* is partly driven by ermine and marten predation, as well as possible raptor predation for which we do not have good data. A similar correlation has been noted in Fennoscandia (Hansson & Henttonen 1988).

3. The population fluctuations of the 3 dominant rodent species are not disappearing as a result of climate change. Both *Myodes* and *Microtus* spp. are increasing in abundance as time progresses, in correlation with increased primary production.

4. Predation and food supply can both be implicated as partial causes of population change in *Myodes* and *Microtus* and are associated with summer population changes in *Peromyscus.* The puzzle is that as small mammal predators have increased, so has rodent population abundance, the opposite prediction that one would expect from predation theory if predation was a major factor causing population changes. The missing factor in the equation is social behavior, which can cause reproductive suppression (Gilbert *et al.* 1986) as well as prevent or increase infanticide (Millar 2007; Eccard *et al.* 2011). These results are consistent with the experimental findings of Johnsen *et al.* (2017).

5. Boonstra and Krebs (2006) suggested that *M. rutilus* population changes were linked to snowshoe hare cycles via nutrient cycling. Our data are inconsistent with this suggestion. With 42 years of data, we have found no significant correlation of snowshoe hare densities with current *Myodes*, *Microtus* or *Peromyscus* densities, nor are any of these rodent densities correlated with hare densities lagged by one or two years. Any short-term correlation between snowshoe hare numbers and those of small rodents we would suspect to be spurious.

There is a general feeling among conservation biologists that biodiversity is being lost on an ever-increasing scale and that most populations that have been studied are declining in abundance (Estes *et al*. 2011; McRae *et al.* 2017; Ripple *et al.* 2017; but see Vellend 2017). Added to this view is the overall observation that climate change is occurring and is most severe in the arctic regions (IPCC 2013). We could expect from these 2 views that we should see ecosystem disruptions in the Kluane boreal forest. Our observations suggest the contrary view. Populations of small rodents in this ecosystem are increasing in overall abundance and we have no information on species losses among these mammals. Vegetation (in particular shrubs) is growing more and in spite of a bark-beetle attack on white spruce trees in the 1990s trees continue to grow and the forest is regenerating (Berg *et al.* 2006; Krebs *et al.* 2014). Dirzo *et al.* (2014) noted that in many ecosystems if large mammals were eliminated or greatly reduced, rodent populations increased. Our data do not fit this paradigm exactly because rodents have increased but large mammals continue to be present at moderate numbers in this Yukon community.

If our data are an anomaly within the global pattern of trophic downgrading, several reasons can be given in explanation. Northern terrestrial ecosystems are particularly slow moving because of the severe winters, and, despite rapid climate change, the growing season is limited to approximately 12 weeks from late May to mid-August. The species in the boreal ecosystem are all post-glacial colonists from approximately 12 000 years ago, when the glaciers receded and recolonization began. As such, they are likely to be nearer the generalist end of the spectrum than specialist species. Perhaps the greatest contrast with more temperate and tropical conservation issues is that terrestrial ecosystems in the Kluane region have had minimal human impact. Resident First Nation peoples have carried out subsistence harvesting for millennia, and while the influx of settlers over the past 120 years has varied with economic booms, total numbers have stayed below 1000. There is no commercial forestry but some firewood gathering, no agriculture industry, no domestic cattle, and a small mining footprint. The analysis by Murray *et al.* (2017) suggests that for the next 100 years the Kluane ecosystem portion of the boreal forest will be the least affected by climate-induced forest fragmentation and loss. As such, our study could be a boreal forest control in which the climate drivers and the human drivers are not confounded.

The largest possible changes we can see in the next 100 years that would greatly disrupt this part of the boreal zone are emergency events such as extensive forest fires or changes in land use that compromise the integrity of intact forests (e.g. widespread logging or road networks). Unless disruptions are very extensive, we can see no problems in ecosystem recovery, with the caveat that the time scale of recovery may be well over 100 years.

We do not wish to project our positive conclusions for small rodents onto the larger species in this ecosystem. The harvest of Canada lynx, grizzly and black bears, moose, caribou, and thin-horn sheep are co-managed by First Nations and other governments with a conservation objective. Given the problems that large mammals face in most parts of the Earth, our area is again a contrast at present (Ripple *et al.* 2014; Boonstra *et al.* 2018) because none of these large mammal species are under threat.

The bottom line of our analysis is very positive for the continuation of this small mammal community in the Kluane region. We know that to be correct because of our long-term monitoring and we stress again that in all ecosystems the "devil is in the details" and these details can only be known by hard work and continued monitoring.

### CONCLUSION

Our 46-year study of these small rodents is a model because it is long-term with a consistent methodology on fixed sites undisturbed except by climate change. We have rigorously documented profound changes that would never have been observed without this long-term effort. It has both given us deep insights and deep puzzles. It has also documented that the signature of climate change is clear in these pristine forests. Despite all our efforts, there are large unknowns in this ecosystem. The birds of prey have not been studied in the continuous detail we would have liked. Great-horned owls are suggested to be declining but we do not know the causes. All the migratory birds are particularly difficult to classify with respect to needed conservation actions and at present we have no data on their abundances. The plant base of the Kluane region is well known but few experimental studies have been done, and less long-term monitoring than is desirable. In consequence our plea is for ecosystem monitoring to follow in the footsteps of climate monitoring with adequate resources so that in future we can construct an analog of the Intergovernmental Panel on Climate Change (IPCC) for the ecosystems of the Earth.

Once there is adequate and continuing funding, there are still 2 unresolved problems in recommending more long-term monitoring studies. One is where to do them, and the other is how long they should be carried forward. The *where* question is most easily answered because you need a protected area to carry out long-term monitoring, although that is not always easy to provide. The *how long* question is more difficult, and the question is whether you are learning new concepts and insights from the continuing work. If you adopt the weather station paradigm, you should never specify an end date because monitoring is an ongoing requirement for environmental management.

Our Kluane monitoring program can be criticized for ignoring the rare rodent species that could be the concern of conservation ecology. From a conservation viewpoint, these rare species are an important part of biodiversity. From an ecosystem viewpoint, rare species are typically of little consequence to community dynamics. If you cannot do everything within your budget, we think it is more important to study the dominant species because they are the key to ecosystem change. For this particular boreal forest ecosystem, the 2 missing elements that need study are the fungi with their mycorrhizal associates and the impacts of climate change on the shrubs and ground vegetation that provide the food base for all of the herbivores. From a regional perspective, the linkage of the alpine tundra ecosystem with the boreal forest ecosystem is an almost unstudied element with consequences that can flow in both altitudinal directions as the climate shifts. There is much left to do and northern Canada provides some of the few ecosystems on Earth that are ruled by climate change rather than by human disturbances.

### ACKNOWLEDGMENTS

We thank Mark O'Donoghue, Elizabeth Hofer, Peter Upton and many students who have worked over the years to capture these measurements. We thank 2 reviewers for critical comments that improved the manuscript. Research funding was provided by the Natural Science and Engineering Research Council of Canada (RB, CK and SB). The facilities of the Kluane Lake Research Station of the Arctic Institute of North America were essential to this long-term research program, and we thank Andy and Carole Williams, Lance Goodwin and Sian Williams for their assistance.

### SUPPLEMENTAL MATERIALS

**Cite this article as:**

## Cite this article as:

Krebs CJ, Boonstra R, Gilbert BS, Kenney AJ, Boutin S (2019). Impact of climate change on the small mammal community of the Yukon boreal forest. *Integrative Zoology* **14**, 528–41.

## Supporting information

Additional supporting information may be found in the online version of this article at the publisher's website.
**Figure S1** Red‐backed voles (*Myodes rutilus*) instantaneous rate of change from autumn to the following spring in relation to winter maximum snow depth, 2000–2018 at Kluane Lake. There is an indication of a weak positive relationship (*R* ^*2*^ = 0.15, *P* = 0.10. Average snow depth over winter is highly correlated with maximum snow depth (*r* = 0.82, *n* = 18).
**Figure S2** Deer mouse (*Peromyscus maniculatus*) autumn population density 1976–2018 at Kluane Lake, with 95% confidence limits. No deer mice were live trapped in the forest between 1990 and 1995 in spite of extensive trapping. These data are discussed in Krebs *et al*. (2018b). Figure updated from Krebs *et al*. (2018b) with permission.

Supporting Information
