# Supplementary material for: Impact of climate change on the small mammal community of the Yukon boreal forest
Source: Integr Zool. 2019 Oct 22;14(6):528–41. doi: 10.1111/1749-4877.12397 (PMC6900156; doi:10.1111/1749-4877.12397)
Supplement: Supplementary file 1 — Supporting Information [file INZ2-14-528-s001.pdf]

Integrative Zoology 2019; 14: 528–541 doi: 10.1111/1749-4877.12397

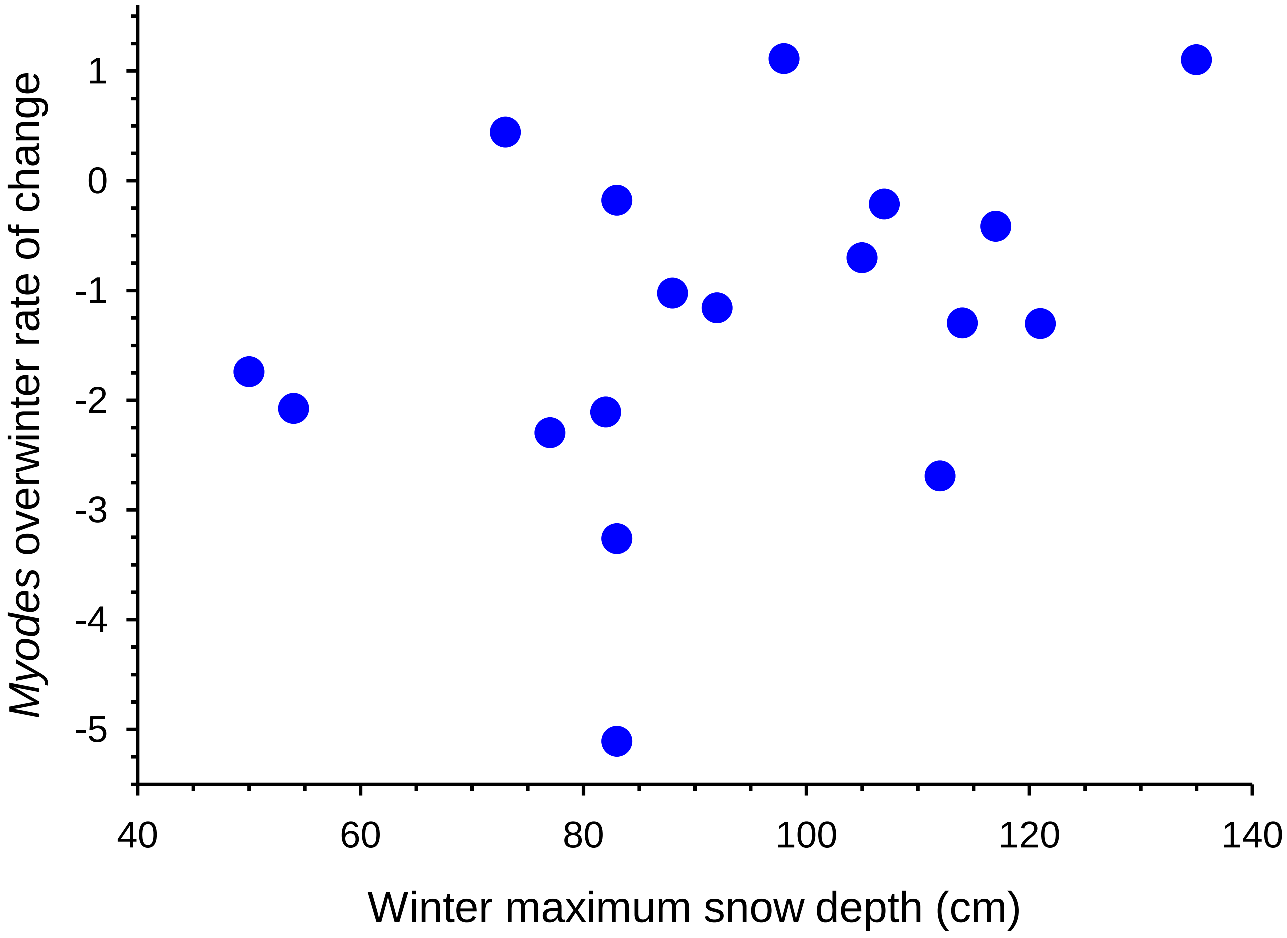

Figure S1 Red-backed voles (*Myodes rutilus*) instantaneous rate of change from autumn to the following spring in relation to winter maximum snow depth, 2000–2018 at Kluane Lake. There is an indication of a weak positive relationship (R^2 = 0.15, P = 0.10. Average snow depth over winter is highly correlated with maximum snow depth (r = 0.82, n = 18).

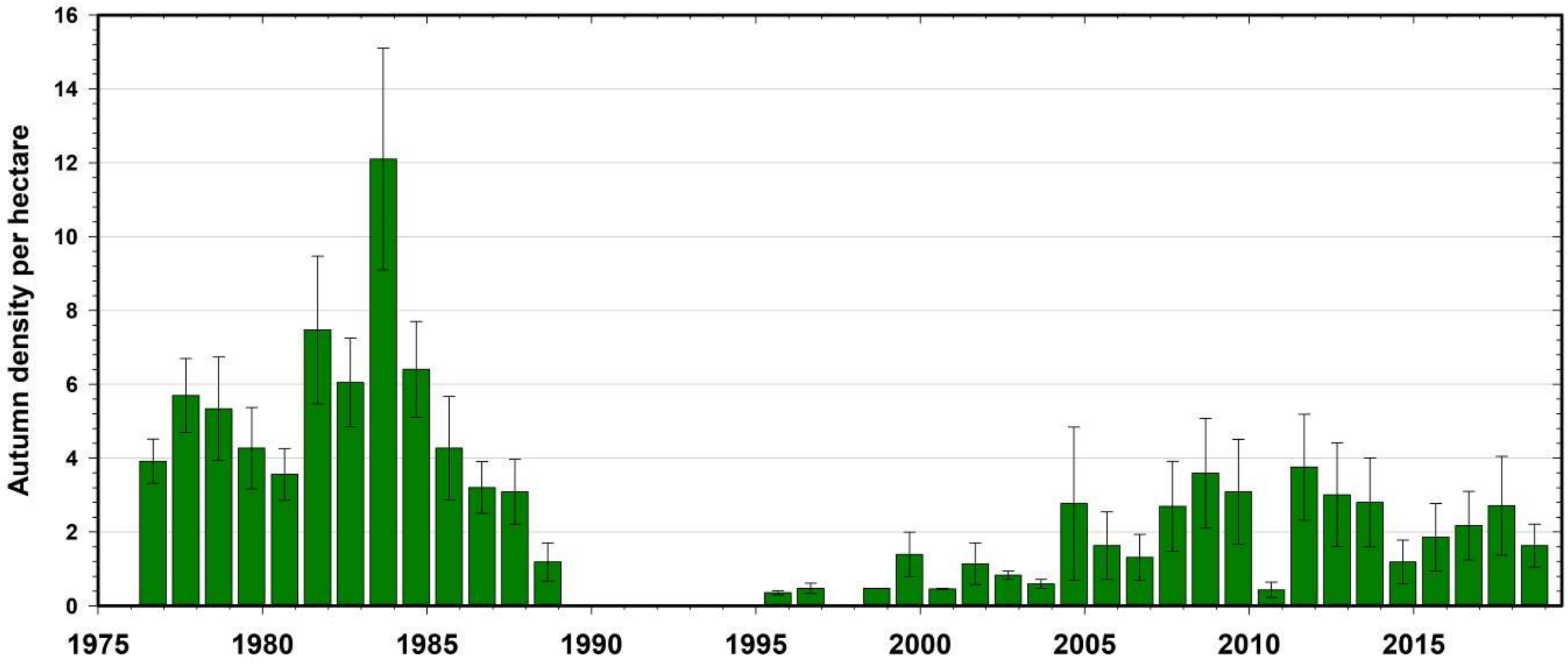

Figure S2 Deer mouse (*Peromyscus maniculatus*) autumn population density 1976–2018 at Kluane Lake, with 95% confidence limits. No deer mice were live trapped in the forest between 1990 and 1995 in spite of extensive trapping. These data are discussed in Krebs *et al*. (2018b). Figure updated from Krebs *et al*. (2018b) with permission.
